# Dynamic attentional modulation of vision across space and time after right hemisphere stroke and in ageing

**DOI:** 10.1016/j.cortex.2012.10.005

**Published:** 2013-07

**Authors:** Charlotte Russell, Paresh Malhotra, Cristiana Deidda, Masud Husain

**Affiliations:** a Centre for Cognition and Neuroimaging, Department of Psychology, Brunel University, Uxbridge, UK; b Laboratorio di Neuropsicologia, Fondazione Santa Lucia IRCCS, Rome, Italy; c Division of Brain Sciences, Imperial College London, London, UK; d Institute of Cognitive Neuroscience, UCL, London, UK; e Nuffield Department of Clinical Neurosciences, University of Oxford, UK; f Department of Experimental Psychology, University of Oxford, UK

**Keywords:** Visual attention, Right parietal cortex, Stroke, Attentional Blink, Ageing

## Abstract

**Introduction:**

Attention modulates the availability of sensory information to conscious perception. In particular, there is evidence of pathological, spatial constriction of the effective field of vision in patients with right hemisphere damage when a central task exhausts available attentional capacity. In the current study we first examined whether this constriction might be modulated across both space and time in right hemisphere stroke patients without neglect. Then we tested healthy elderly people to determine whether non-pathological ageing also leads to spatiotemporal impairments of vision under conditions of high attention load.

**Methods:**

Right hemisphere stroke patients completed a task at fixation while attempting to discriminate letters appearing in the periphery. Attentional load of the central task was modulated by increasing task difficulty. Peripheral letters appeared simultaneously with the central task or at different times (stimulus onset asynchronies, SOAs) after it. In a second study healthy elderly volunteers were tested with a modified version of this paradigm.

**Results:**

Under conditions of high attention load right hemisphere stroke patients have a reduced effective visual field, over a significantly extended ‘attentional blink’, worse for items presented to their left. In the second study, older participants were unable to discriminate otherwise salient items across the visual field (left or right) when their attention capacity was loaded on the central task. This deficit extended temporally, with peripheral discrimination ability not returning to normal for up to 450 msec.

**Conclusions:**

Dynamically tying up attention resources on a task at fixation can have profound effects in patient populations and in normal ageing. These results demonstrate that items can escape conscious detection across space and time, and can thereby impact significantly on visual perception in these groups.

## 1 Introduction

Our eyes are bombarded with a vast amount of information from across the visual field. Visual acuity for this information can be mapped by standard perimetry. However, what is available to conscious perception is affected by factors other than low-level visual processes. Availability of attentional resources appears to be critical for awareness (e.g., see, Lavie, 2005; Rees et al., 1997, 1999; Schwartz et al., 2005; Vanni and Uutela, 2000). If the amount of attention required for a task at fixation is high, there is an effective constriction of the available visual fields and failure to perceive otherwise salient onsets in healthy people (Russell et al., 2004). The dynamic loss of vision for peripheral targets when attentional resources are occupied can be seen by the decrease in neural activity for peripheral checkerboard patterns even in early visual cortex when task demands at fixation are high (Schwartz et al., 2005 see also, Rees et al., 1997).

Recently O'Connell et al. (2011) examined the effect of central attentional load on spatial orienting towards peripheral events, measuring event-related potentials to assess timing of the modulation. The early N1 signal (previously shown to indicate enhanced attentional processing) was attenuated, particularly over the right hemisphere, for expected peripheral targets when participants completed a high load task at fixation. Modulation of N1 is consistent with evidence linking this signal to the right temporo-parietal cortex. The key role of these regions in directing attention is well documented (e.g., Corbetta and Shulman, 2002; Friedrich et al., 1998). Indeed fMRI has revealed modulation by load in these regions, particularly right intra-parietal sulcus, suggesting an important contribution to non-spatial attentional capacity (e.g., Culham et al., 2001).

Compatible with studies on healthy participants, damage to the right hemisphere leads to impairments in attention. Visuospatial neglect, frequently occurring after damage to right parietal cortex (e.g., see, Driver and Mattingley, 1998; Mort et al., 2003; Vallar, 2001), is characterized by a loss of awareness for items in the visual field contralateral to the lesion. Although the most salient features of neglect involve spatial attention, as deficits are strongly lateralized, there is evidence that non-spatial components of attention are affected (see Husain and Rorden, 2003; Robertson, 2001). These patients have problems in sustaining attention over minutes (e.g., Malhotra et al., 2009: Robertson et al., 1997) and increasing alertness ameliorates the lateralized symptoms (e.g., Chica et al., 2012; Degutis and Van Vleet, 2010; Thimm et al., 2006; Robertson et al., 1998). Further, non-spatial attention capacity deficits in these patients affect conscious awareness for items across the visual field. Vuilleumier et al. (2008) examined responses to background checkerboards in early visual cortex of neglect patients completing a task at fixation. When central task load was low, early visual cortex responded to the checkerboards on both sides. However, when central load was increased, responses to checkerboards presented to the left visual field were reduced or abolished (see also, Bonato et al. (2010); Peers et al., 2006; Sarri et al., 2009). Russell et al. (2004) revealed that patients with damage to right parietal cortex, even without neglect, missed peripheral targets when they were required to complete a difficult task at fixation. Performance was particularly poor on the contralesional side but there was even loss of ipsilesional vision when central task demand was sufficiently high.

In addition to spatial impairments in conscious awareness under high load, observers can suffer detection deficits over *time*. The ‘Attentional Blink’ (AB) paradigm is used to delineate temporal capacity limits to perception (Raymond et al., 1992; Shapiro et al., 1994). Participants are presented with two targets embedded in a stream of rapidly presented items at fixation. Healthy young participants often fail to detect the second target if it is presented within a short lag of the first (under ∼500 msec). The time taken to process the first target occupies capacity, rendering it briefly difficult to identify another target; indeed task load manipulations within the AB paradigm indicate that perception of the second target reflects current availability of attentional resources (e.g., Elliott and Giesbrecht, 2010). Patients with visuospatial neglect have shown an extended ‘AB’, with a failure to report second targets over a much longer lag period (e.g., up to 1300 msec) (see Husain et al., 1997; Hillstrom et al., 2004; Rizzo et al., 2001). However, it is unclear whether such deficits can also be protracted spatially, particularly to the contralesional side, as previous studies have used centrally presented targets. Our first study aims to assess whether the spatial contralesional deficit for discriminating stimuli when performing a demanding central task extends temporally and impairs perception for a longer period.

This potential attention-modulated loss of available visual field – over space and time – is also relevant to healthy ageing and our understanding of the impact of age-related decline on daily function. Investigators have developed tests of the Useful Field of View (UFOV) and correlated performance with driving ability (e.g., Clay et al., 2005; Owsley et al., 1995). UFOV tests typically involve making judgements on a central item whilst attempting to discriminate peripheral items, often with concurrent distractors. Older adults who, despite having intact visual fields, are poor at this test are more dangerous drivers as indexed by measures including road accidents and driver simulator performance (Clay et al., 2005). Crucially, these studies have not modulated the amount of attention required in the central task in order to examine how this impacts on deployment of attention to peripheral items. Some investigations have also reported that older participants might suffer from an AB that is longer and of greater magnitude (e.g., Georgiou-Karistianis et al., 2007; Maciokas and Crognale, 2003), but no studies have examined perception across the visual field in these paradigms. In our second experiment, we used our paradigm to probe deployment of attention over space and time within healthy ageing when participants perform a demanding task at fixation.

## 2 Experiment 1

### 2.1 Method

#### 2.1.1 Participants

**Fig. 1:**
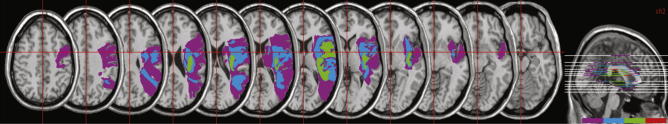
Lesion overlap showing regions affected in 4 of the participating patients. The greatest areas of overlap were in the sub-cortical white matter (in green) with a very small focus (in red) where all four patients had a common region of damage (MNI coordinates 28, −26, 24).

Five patients with right hemisphere stroke participated in the study. Patients were aged from 55 to 75 (mean 66 years). All were in-patients at the Fondazione Santa Lucia Neuro-Rehabilitation Hospital in Rome, Italy. They had suffered from their stroke on average 12 weeks prior to entering the research programme. Brain lesions, imaged by CT or MRI, were reconstructed with MRICro software (http://www.sph.sc.edu/comd/rorden/mricro.html), plotted with the use of a graphics tablet (WACOM Intuos A4). See Fig. 1 for lesion mapping images, which demonstrate widespread involvement including frontal and parietal regions. Scans were unavailable for one patient (the radiology report stated that there was damage to right frontal, parietal and temporal regions affecting cortical and sub-cortical structures).

None of the patients suffered from neglect at the time of testing according to a standard clinical examination. All patients had intact visual fields as tested by confrontation, 4/5 patients had constructional apraxia as revealed by performance on the Rey–Osterrieth complex figure and block design of the Wechsler Adult Intelligence Scale. Patients were compared with five age-matched healthy control participants. Their ages ranged from 56 to 70 (mean 65 years), all reported normal/corrected to normal vision. All participants gave written informed consent according to the Declaration of Helsinki. The study was approved by both the hospital and university research ethics committees.

#### 2.1.2 Apparatus & stimuli

**Fig. 2:**
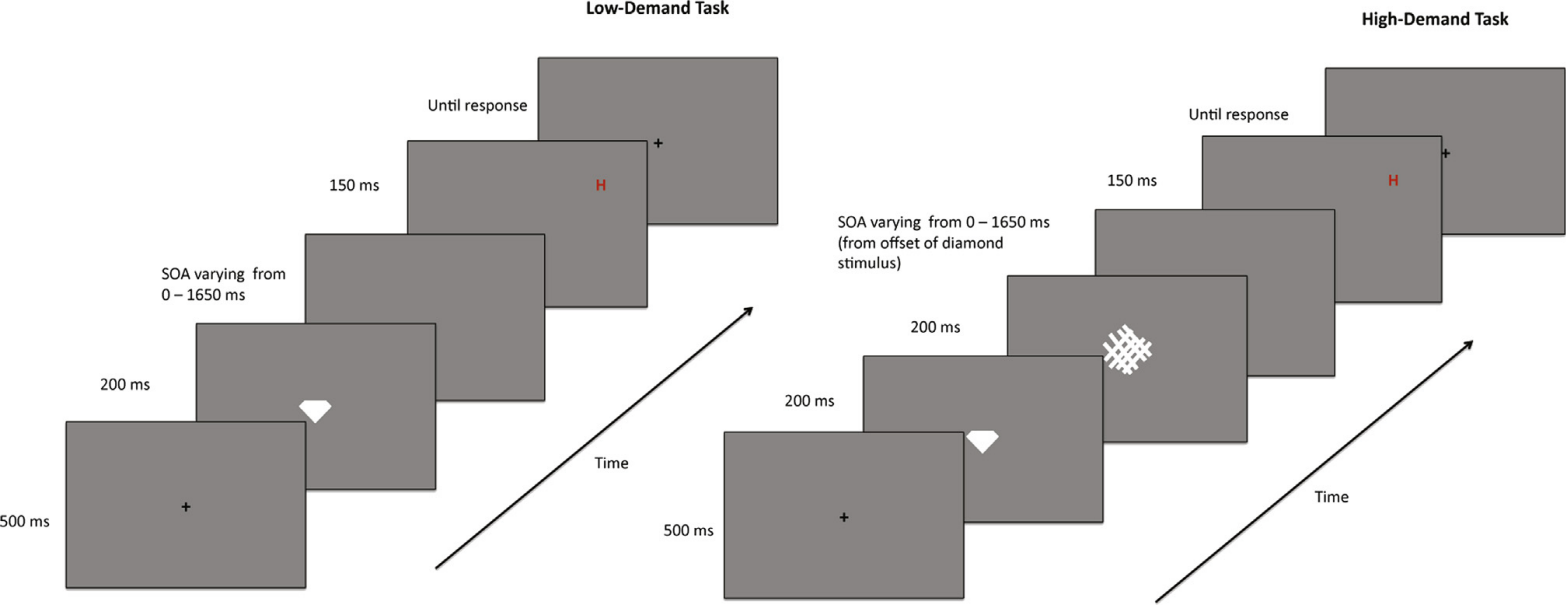
Schematic example of trial events for both the low attention demand (on the left of the figure) and the high attention demand (on the right) trials. For clarity of the small stimuli the figures display only the central part of the screen.

The experiment was programmed with Psyscope software (Cohen et al., 1993) run from a Macintosh G4 laptop computer. A small white diamond shape (1° across, see Fig. 2) was presented at fixation with either its top or bottom apex missing. During the low load condition only the diamond was presented in the centre. In the high load task, a mask stimulus appeared immediately after the diamond was extinguished to increase demand.

On each trial a red upper case letter appeared elsewhere on the screen (either an H or a T). Possible positions of these letters were at one of the four corners of two imaginary squares centred on the diamond. The eccentricity of imaginary square corners could be near to the diamond (2°) or further (6°). Size of the letters varied according to peripheral distance, with those further away scaled account for the cortical magnification factor of items nearer the fovea. Those at 2° were .46° across those at 6° were .69° across. There were an equal number of near and far letters presented and they were distributed approximately equally across the four peripheral directions. Stimuli were displayed on a mid-grey background.

#### 2.1.3 Procedure

Trials began with a central fixation cross presented for 500 msec, followed by the diamond stimulus for 200 msec. In high load blocks, the mask stimulus appeared immediately afterwards for 150 msec. A letter was presented in the periphery in every trial. Letter presentation was either simultaneous with the central diamond or delayed. During stimulus onset asynchrony (SOA) trials there were three possible asynchronies (450 msec, 850 msec and 1650 msec). Simultaneous letter trials were in separate blocks. Differing SOAs were presented randomly, with an approximate equal number of each type across the blocks. There were four types of experimental block: Low-demand, simultaneous letter presentation; Low-demand, SOA letter presentation; High-demand, simultaneous letter presentation; High-demand, SOA letter presentation.

Most participants completed 10 experimental blocks. Two blocks each of Low-demand and High-demand simultaneous letter blocks and three blocks each of Low-SOA and High-SOA. Each block had 50 trials. Participants completed these blocks in two to three separate 1-h sessions. Presentation order of the blocks was counterbalanced. Task instructions emphasized the need to complete the central task accurately.

Participants sat approximately 50 cm from the computer screen and made verbal responses, stating first whether the diamond was missing the top or bottom apex and second what they believed the identity of the letter to be. Two experimenters were present throughout testing. One sat facing participants with the response button box, enabling them to cancel trials in which participants moved their eyes from screen centre and to enter verbal responses. The other started each block, explained the task and observed whether the participant appeared to understand task requirements.

## 3 Results and discussion

### 3.1 Analysis across groups

**Fig. 3:**
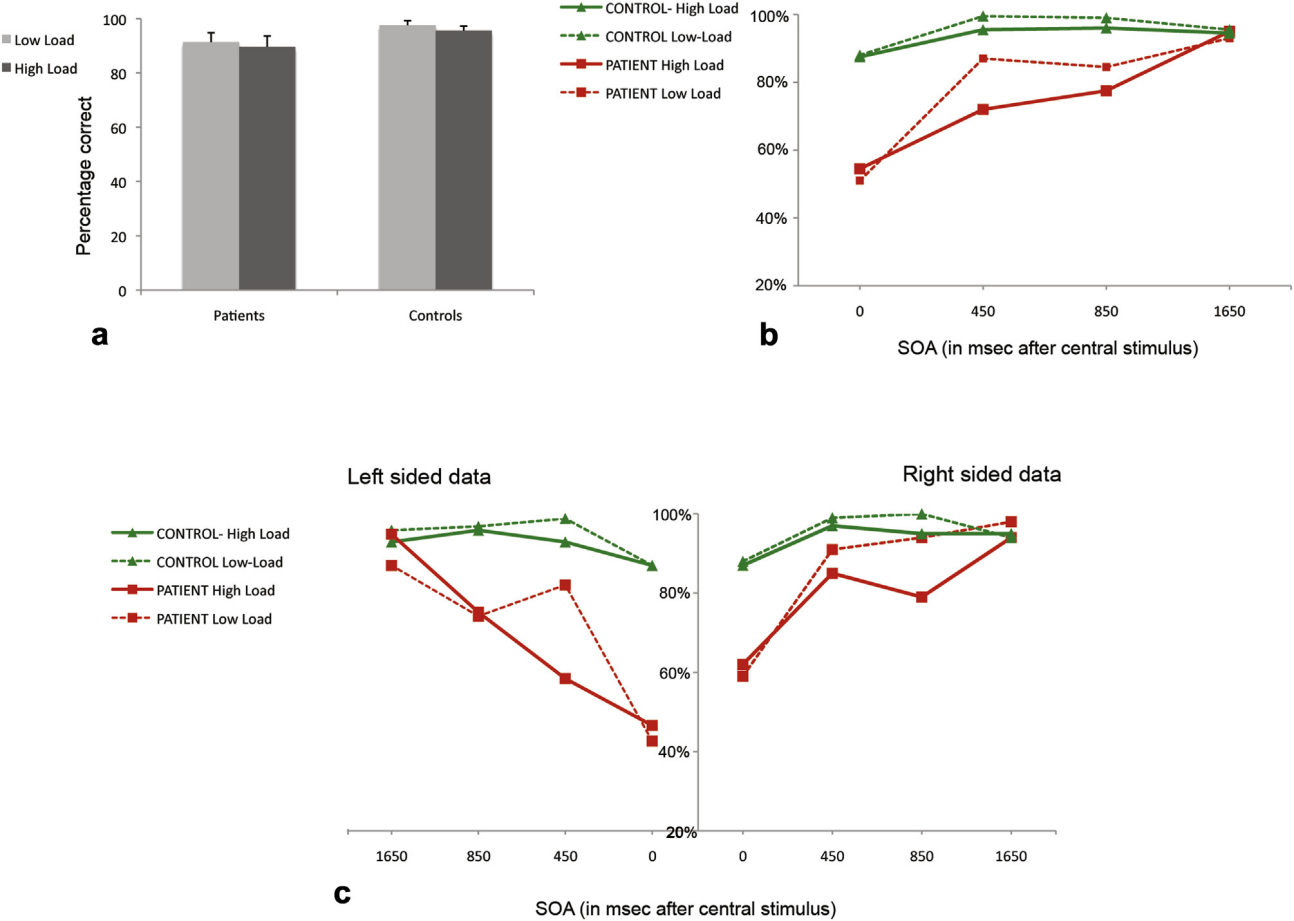
Results for Experiment 1. 3a displays performance for the central fixation task for both groups across both levels of load. 3b gives the mean percentage of correct discriminations of peripheral letters collapsed over both distance from fixation and side of presentation. 3c shows peripheral discrimination data split by side of presentation. Left-sided stimuli are to the left of the central *y*-axis and right-sided span out to the right.

First, performance on the central diamond task was examined (see Fig. 3a for this data). This revealed participants to be equivalently accurate across both experimental groups for each level of attentional demand [interaction between task load and group was not significant; *F* (1, 8) < 1]. Thus participants fulfilled instructions and gave sufficient priority to performing the central task. To assess the consequences of this on deployment of attention to other locations, we examined participants' discrimination of peripheral letters (Table 1a and b).

An ANOVA was conducted with four within-subjects factors: SOA (0 msec; 450 msec; 850 msec; 1650 msec); load of central task (high or low); side of peripheral stimulus (left or right) and distance of peripheral stimulus (near or far) and the between-subjects factor of group (patient or control). Results revealed significant interactions between both SOA and group [*F* (3, 7) = 10.775, *p* < .01], as well as between side of peripheral letter and group [*F* (3, 7) = 9.627, *p* < .01]. Crucially there was an interaction between SOA, load, side and group [*F* (3, 7) = 3.611, *p* < .05], indicating that patients and controls were differentially affected by manipulations of SOA, the load of the task and the side of space that the letter was presented. Fig. 3b gives the data collapsed over both side and distance of letter stimuli.

The control group's letter discrimination ability whilst completing the central task remained robust across both load conditions and all SOAs, but the patient group's performance was lower for the first three SOA's (0 msec, 450 msec, 850 msec) and lower again whilst completing the more difficult central task. Presumably due to successful correction for cortical magnification factors, no comparisons involving the distance of peripheral stimuli reached significance. Therefore, for simplicity, data were collapsed across distance in further analyses.

The significant effect of the factor of side in the ANOVAs above suggests differences in the perception of left versus right peripheral stimuli. This is potentially very important and so the data were split according to side of letter presentation and re-analysed separately (Fig. 3c).

For stimuli on the left, ANOVA revealed significant interactions between SOA and group [*F* (1, 9) = 6.705, *p* < .01] as well as for the crucial comparison of SOA, load and group [*F* (3, 7) = 4.006, *p* < .05]. In contrast analysis for right-sided letters revealed a main effect of SOA and group [*F* (1, 9) = 6.046, *p* < .01] but, importantly, no interaction between SOA, load and group [*F* (3, 7) < 1].

Independent sample *t*-tests on the data in Fig. 3c revealed that whereas for left-sided stimuli patients and controls significantly differed in accuracy at both load levels at 0 msec [*t* (9) = −4.412, *p* < .01 and *t* (9) = −5.109, *p* < .01 for low and high respectively] and 450 msec [t (9) = −3.356, *p* < .05 and *t* (9) = −5.634, *p* < .01 for low and high respectively], at higher SOAs the groups' scores were not significantly different. For right-sided stimuli, between subjects *t*-tests revealed that only data for 0 msec significantly differed between the groups [*t* (9) = 6.691, *p* < .01 during low load and *t* (9) = 6.057, *p* < .01 for high load].

The patient group was impaired in reporting peripheral letters compared to controls when these letters appeared simultaneously or within a short delay period from the central stimuli. This effect appeared to be modulated by available attentional capacity, as discrimination was worse when they were required to complete a more demanding task at screen centre. This pattern was prominent for letters appearing on the left side of space as there was a significant interaction between task demand, SOA condition and group for these stimuli. However, even on the right side, right-hemisphere patients were less accurate than controls when letters appeared simultaneously with the central diamonds.

### 3.2 Analysis of patient data

An initial ANOVA involving within-subjects factors of SOA (4 levels), load (2 levels) and side (left *vs* right) revealed significant main effects of SOA and side [*F* (3, 7) = 23.94, *p* < .001 and *F* (1, 9) = 9.607, *p* < .05 respectively]. In addition, there was a significant interaction between SOA, load and side [*F* (3, 7) = 5.069, *p* < .05]. Again, to investigate differential responses according to side, separate analysis was carried out for letters appearing on the left and right. On the left there was a critical interaction between SOA and load [*F* (3, 7) = 5.289 *p* < .05). In contrast discrimination accuracy for letters on the right did not reveal this interaction (*F* (3, 7) < 1, n.s.].

Further analysis of left-sided performance was carried out. Of interest here were differences in discrimination according to load at the various SOAs. For left-sided stimuli during the low-demand condition, there was a significant difference in detection between the 0 msec and 450 msec condition [*t* (4) = −5.14, *p* < .01], which was not the case during the high demand condition [*t* (4) = −1.403, n.s.]. This pattern continues for stimuli at 850 msec, as during the low load task, patients detected significantly more letters than those presented simultaneously [*t* (4) = −3.382, *p* < .01]. By contrast, when they were completing the high load task patients *still* did not detect significantly more than at 0 msec [*t* (4) = −1.863, n.s.]. At 1650 msec, discrimination was significantly better than for letters presented simultaneously with the central task for *both* levels of central task load: *t* (4) = −10.874, *p* < .001; *t* (4) = −7.071, *p* < .01 for low and high load respectively.

Vision across the contralesional field in this group of patients appears critically impaired when they complete an attentionally demanding task at fixation. Crucially this impedance is not solely at the time the central task is presented but extends forward in time to give a “spatial attentional blink” on the contralesional side lasting for up to 850 msec. These patients do not suffer from visuospatial neglect-however the lesions from which they suffer appear to reduce attentional capacity such that loading processing resources at fixation causes both a spatial *and* temporal loss of visual perception.

## 4 Experiment 2

Patients in the previous study were compared to healthy age-matched participants. However, there is evidence to suggest that completing a task at fixation constricts the available field of peripheral vision even in older participants (e.g., Owsley et al., 1995). We hypothesized that if the level of attention required in the task described in Experiment 1 was increased, older participants might begin to show a failure to discriminate peripheral stimuli. The paradigm developed in the first study lends itself well to examining whether any impairments older people have in reporting peripheral events (Owsley et al., 1995) interact with the lengthened attentional blink described by other authors in elderly individuals (e.g., Maciokas and Crognale, 2003; Georgiou-Karistianis et al., 2007).

### 4.1 Method

As we were no longer assessing impairments in stroke patients but differences between healthy younger and older groups, the methodology of Experiment 1 was manipulated to increase difficulty. First, display time of both peripheral letters and central diamonds was shortened to 150 msec (from 200 msec in the first study). Second, peripheral letters were no longer red but were now white. Finally, the SOAs differed so that letters appeared at either 0 msec, 250 msec, 450 msec, 850 msec from the central diamond stimulus. All other methodological details were identical.

#### 4.1.1 Participants

A group of 21 healthy participants aged from 52 to 78 years of age (mean: 63 years) were compared to a group of 10 younger participants aged from 19 to 24 years (mean: 21 years). Ethical approval for the study was given by the university research ethics panel.

### 4.2 Results

#### 4.2.1 Analysis comparing older and younger groups

**Fig. 4:**
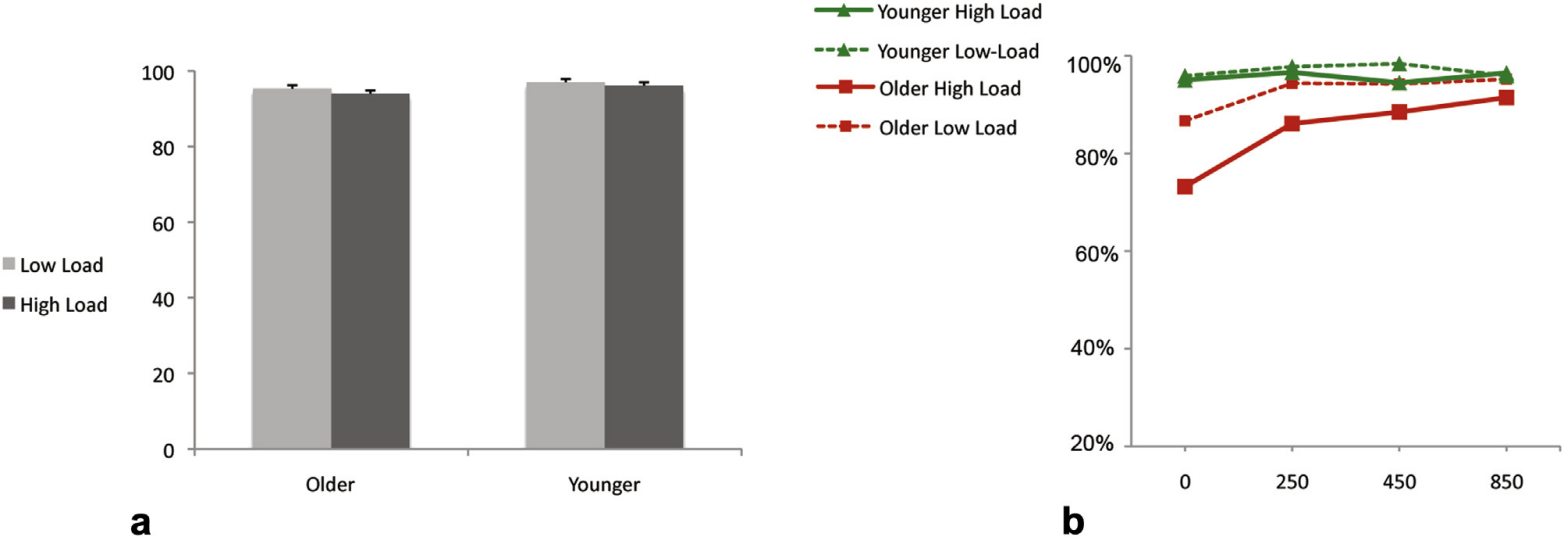
Results for Experiment 2. 4a displays central task performance for the two groups across both levels of load. 4b gives the means percentage of correct discriminations of peripheral letters collapsed across both distance from fixation and side of presentation.

Examination of performance on the central task confirmed that accuracy was high and equivalent across participant groups and conditions (Fig. 4a). There was no significant interaction between the within-subjects factor of task load and the between-subjects factor of group [*F* (1, 30) < 1, ns].

An initial ANOVA was carried out with the within-subjects factors of SOA (zero, 250 msec, 450 msec, 850 msec), central load (high *vs* low), side of letter presentation (left *vs* right) and the between-subjects factor of age group (older *vs* younger). There was no interaction between group and side [*F* (1, 30) = 2.38, *p* = .14] and data were subsequently collapsed across side of presentation. Analysis did reveal significant interactions between load and group [*F* (1, 30) = 7.38, *p* < .05], as well as between group and SOA [*F* (3, 29) = 6.63, *p* < .001]. See Fig. 4b and Table 2a and b.

Due to the interaction between load and group, data were split and additional ANOVAs were performed on data from the low and high load tasks. First, during the high load central task, there was a significant interaction between group and SOA [*F* (3, 28) = 5.30, *p* < .01]. This contrasts with the low load condition as there was no significant interaction between SOA and group [*F* (3, 28) = 2.10, n.s.]. Attentional demand of the central task appears critical to differences between performance across the age groups. Independent subject *t*-tests examined these differences between group performances. During the high-demand task older participants were significantly worse than the younger group at each SOA [*t* (28) = −3.33, *p* < .01; *t* (28) = −3.77, *p* < .01; *t* (28) = −2.34, *p* < .05; *t* (28) = −2.9, *p* < .05 for zero, 250 msec, 450 msec and 850 msec respectively]. Whereas in the low-load task although zero and 250 ms did differ [*t* (28) = −2.39, *p* < .05; *t* (28) = −2.13, *p* < .05 respectively] there was no longer a significant loss of accuracy for the older group at 450 msec [*t* (28) = −1.84, ns] and 850 msec [*t* (28) = −.33, n.s.].

#### 4.2.2 Analysis of older group

An ANOVA on SOA (4 levels) and load (2 levels) revealed highly significant main effects of both SOA [*F* (3, 28) = 19.83, *p* < .0001] and load [*F* (1, 30) = 22.73, *p* < .0001] and a significant interaction between the two [*F* (3, 28) = 4.14, *p* < .01].

Paired samples *t*-tests further investigated the source of this interaction. In the low load task the discrimination performance of older participants did not significantly differ between the three SOAs [all *t* (20)< 1, n.s.]. Whereas during the high load task, performance was equivalent at 250 and 450 msec [*t* (20) = −1.34, n.s.], but at 850 msec it was significantly better than at either of the two other delays [*t* (20) = −3.17, *p* < .01 and *t* (20) = −2.42, *p* < .05 for 250 msec and 450 msec respectively].

The results described here provide new evidence that perception of older individuals is strongly impaired when they are required to pay attention to a task at fixation. Compared to younger participants, those in the older group were far less accurate in discriminating peripheral letters not only when presented simultaneously with the central diamonds but for a delay period afterwards. This is the first evidence of a “spatiotemporal” attentional blink across the visual field modulated by the demand of a primary task at fixation in older healthy participants.

## 5 General discussion

The experiments presented here reveal the spatial and temporal consequences to the effective visual field of an attention-demanding task at fixation. Experiment 1 demonstrated that patients with right hemisphere damage, but without visuospatial neglect, were severely impaired in discriminating letters even near to fixation whilst maintaining a high level of accuracy for the primary task. Spatially, this impacted on perception on the contralesional side. Temporally, this impact lasted well beyond the presentation of central stimuli. Experiment 2 modified the difficulty of the task in order to investigate the effect of healthy ageing on these perceptual effects. This study revealed a significant impairment in older participants, compared to a younger group, in detecting peripheral letters when attention demands to perform the central task was high. Again, this impairment was for items near to fixation and lasted for a lag period after central task presentation. Crucially this was not the case for younger participants. A parsimonious explanation for these results might be that there is a pathological attentional capacity deficit in right hemisphere patients and a decline in attentional capacity as we age. These results are the first demonstration both of a pathological spatiotemporal AB in patients with right hemisphere damage and of the perceptual results of a decline in attention capacity during healthy ageing. The paradigm developed here has revealed itself to be robust and adaptable to different participant groups for the exploration of interactions between spatial and temporal attentional processes. Here, we have been able to show that patients with right hemisphere damage are severely impaired at identifying letters appearing away from a central task. In fact they detect and discriminate only around 50% of these letters at both levels of central task difficulty when they appear simultaneously. This poor performance for letters appearing simultaneously with the diamond task is not simply for those on the contralesional side but also for those presented ipsilesionally (only 60% of these are detected during the high load task, see Fig. 3c). However, the critical aim of this study was to examine whether difficulties in discriminating the letters extended temporally. That is, if the peripheral letters appear after the central diamonds, is there a protracted period over which discrimination remains poor? Further, is this posited lag period affected by the attentional demand of the central task?

Our results demonstrate that, when there was a high attention demand in the central task, patients were impaired in accurately responding to these letters for a lag period that lasted for up to 850 msec. They failed to accurately discriminate significantly more letters at an SOA of 850 msec than when these letters were simultaneously presented with the diamonds. Critically, although patients and controls demonstrate very different performance in their perception away from fixation, performance of both groups for the central task, at both levels of attentional demand, was equivalent. Therefore, there was not a generalized loss of ability but rather specific failures, revealed both spatially and temporally, in secondary task completion when a large amount of attention was required in a central task. There is effectively less visual field available and so fewer letters are correctly identified away from fixation; we did not find a near versus far effect.

The results of Experiment 1 align well with previous research on similar patients who have shown that increasing the amount of attention required in a central task increases the ipsilesional bias (e.g., Peers et al., 2006) and decreases neural activity for contralesional stimuli (e.g., Vuilleumier et al., 2008). Here we extend this to examine the temporal dynamics of these phenomena, revealing that the increased ipsilesional bias and loss of perception on the contralesional side extends forward in time.

The patients tested here all had suffered from right hemisphere lesions. The majority of them had cortical damage, involving parietal cortex (4/5 patients). The maximal area of overlap was found to be sub-cortical. This interesting finding is consistent with recent research, which has outlined the previously overlooked role of white matter tracts in the neural attention network (e.g., Thiebaut de Schotten et al., 2011, 2005; Doricchi et al., 2008). Tentatively this suggests that damage to a frontoparietal network might lead to the loss of attentional capacity resulting in these findings.

Behaviourally, although most of these patients had suffered from visuospatial neglect at first admission, it is important to emphasize that they no longer clinically suffered from this disorder. The majority (4/5) suffered from more subtle non-lateralized visuospatial deficits, such as constructional apraxia, which can be associated with trans-saccadic deficits (see Russell et al., 2010) but has not previously been associated with the spatiotemporal impairments we have reported here. The findings presented here provide further information on the role of the right hemisphere networks, including white matter, involved in deploying attention. While the research focussing on the neglect syndrome is important, it is also useful to examine patients who no longer have this condition, but nevertheless continue to suffer from attention impairments.

In Experiment 2, we modified our paradigm to examine potential spatial and temporal effects of attention loss in healthy ageing individuals. The results confirmed that, although older participants were able to complete the central task as accurately as younger individuals, when this task demanded more attention their ability to discriminate letters, even in the near periphery, was severely impaired. This impact on perception lasted for up to 450 msec, indicative of an AB for these stimuli, on both sides of space. At low-demand conditions there was little difference between the groups. However, the results changed dramatically when demand on the central task was higher as the healthy older individuals suffered significant loss in the ability to discriminate letters when they appeared simultaneously, 250 msec or 450 msec from the diamond stimuli. This effect of age on spatiotemporal attention has not previously been shown. Although there is evidence of an extended AB with increasing age (e.g., Georgiou-Karistianis et al., 2007) and a central task seems to lead to a reduction in the visual field available away from fixation (e.g., Owsley et al., 1995) evidence of interaction between attentionally modulated spatial and temporal deficits in the effective visual field is demonstrated here for the first time. The finding has important ‘real world’ implications with respect to performance of daily tasks such as driving. Importantly, considering the strong effect of increasing attention load on older participants, it is possible that some UFOV assessments might even underestimate deficits in the available visual field when attention demand at fixation is high.

Although here we are concerned with behaviour, the effects of age on the healthy brain have recently received much research attention. It is well established that the prefrontal cortex undergoes structural and also seemingly functional change with increasing age (see Grady, 2008 for review). Less established are effects on parietal cortex and the right hemisphere white matter underlying these regions. However, it appears to be the case that older participants have significantly more activity in posterior parietal cortex whilst attending to an attentional cue (Jimura and Braver, 2010) and a general greater recruitment of these regions in other attention tasks (Grady, 2008). The authors propose that this age group is less efficient at utilizing attention, possibly as a result of loss of capacity (Jimura and Braver, 2010). Structurally, there is evidence of both cortical parietal atrophy (Bergfield et al., 2010) as well as age-related white matter hyperintensities in this region (Murray et al., 2010). Results found here correspond well with these recent neuroimaging studies as we demonstrate the behavioural consequences of age related degeneration of attentional networks.

The results outlined within this paper are important with respect to the groups studied here but beyond that the paradigm itself is a significant development. Our own previous research using a similar paradigm revealed that if task load is high enough even young healthy participants can miss items in the near periphery (Russell et al., 2004 see Lavie, 2005). Further adaptation of the basic method could be used to investigate attentional capacity across diverse groups such as those with left hemisphere damage or suffering from dementia, enabling the identification of the key brain regions and networks for integration of spatial and temporal components of attention.

In conclusion, we have examined spatiotemporal attention processing capacity in two groups. The first (Experiment 1) consisted of patients with right hemisphere lesions, without neglect. Compared to their healthily ageing counterparts, these individuals suffer from a pathological loss of ability to discriminate simple stimuli even in the near periphery when they complete an unrelated task at screen centre. This loss is modulated by the amount of attention they must give the central task and temporally extends for a period of 850 msec. Secondly (Experiment 2), task modulations made it possible to examine the effects of healthy ageing on visual attention. Here we were able to show that an older group (mean age: 63 years) was as efficient as a much younger group when little attention was required at screen centre. However, they were greatly impaired across the visual field when they were required to allocate more attention centrally. They failed to discriminate simple letters and suffered from an AB of 450 msec. These important results provide a timely demonstration of the importance of visual attention both spatially and temporally for conscious perception and efficient completion of even seemingly undemanding tasks.

## Funding

This work was supported by a European Commission Marie Curie Intra-European Fellowship (011457) and a Brunel Research Initiative (BRIEF) Award to CR and a Wellcome Trust Senior Fellowship to MH.

## Figures and Tables

**Table 1:** a: Patient group means and standard deviations (in brackets) for Experiment 1. b: Control group means and standard deviations (in brackets) for Experiment 1.

	Low central load				High central load			
Left		Right		Left		Right	
Near	Far	Near	Far	Near	Far	Near	Far
**a**								
Zero	.44 (.17)	.41 (.16)	.64 (.12)	.56 (.17)	.48 (.08)	.46 (.23)	.53 (.05)	.71 (.15)
450 msec	.86 (.17)	.80 (.12)	.95 (.05)	.87 (.22)	.48 (.18)	.69 (.19)	.83 (.24)	.87 (.25)
850 msec	.82 (.17)	.68 (.27)	.92 (.10)	.98 (.05)	.83 (.37)	.69 (.29)	.72 (.30)	.87 (.29)
1650 msec	.95 (.74)	.81 (.17)	.98 (.04)	.98 (.05)	1 (0)	.91 (.12)	1 (0)	.88 (.17)
**b**								
Zero	.94 (.11)	.90 (.14)	.87 (.17)	.88 (.16)	.83 (.15)	.91 (.09)	.85 (.15)	.83 (.12)
450 msec	1 (0)	1 (0)	.99 (.02)	1 (0)	.90 (.11)	.98 (.03)	.97 (.05)	.98 (.05)
850 msec	.97 (.06)	.99 (.03)	1 (0)	1 (0)	.99 (.03)	.95 (.10)	.98 (.05)	.93 (.08)
1650 msec	1 (0)	.94 (.13)	.96 (.09)	.91 (.13)	.92 (.08)	.95 (.09)	.94 (.10)	.96 (.09)

**Table 2:** a: Older group means and standard deviations (in brackets) for Experiment 2. b: Younger group means and standard deviations (in brackets) for Experiment 2.

	Low central load		High central load	
Near	Far	Near	Far
**a**				
Zero msec	.88 (12.94)	.86 (13.13)	.76 (21.68)	.71 (19.49)
250 msec	.96 (5.98)	.93 (7.22)	.89 (9.87)	.84 (10.20)
450 msec	.96 (6.49)	.93 (9.58)	.90 (10.47)	.86 (9.15)
850 msec	.96 (6.54)	.95 (7.26)	.92 (7.24)	.90 (11.07)
**b**				
Zero msec	.95 (4.66)	.97 (3.72)	.95 (8.63)	.95 (5.41)
250 msec	.97 (4.44)	.99 (2)	.97 (4.27)	.97 (4.36)
450 msec	.99 (2.44)	.98 (3.04)	.94 (5.83)	.95 (8.23)
850 msec	.97 (4.08)	.95 (8.19)	.96 (6.54)	.95 (7.26)
